# An advanced computing scheme for the numerical investigations of an infection-based fractional-order nonlinear prey-predator system

**DOI:** 10.1371/journal.pone.0265064

**Published:** 2022-03-21

**Authors:** Zulqurnain Sabir, Thongchai Botmart, Muhammad Asif Zahoor Raja, Wajaree Weera

**Affiliations:** 1 Department of Mathematics and Statistics, Hazara University, Mansehra, Pakistan; 2 Faculty of Science, Department of Mathematics, Khon Kaen University, Khon Kaen, Thailand; 3 Future Technology Research Center, National Yunlin University of Science and Technology, Douliou, Yunlin, Taiwan, R.O.C; University of Porto Faculty of Engineering: Universidade do Porto Faculdade de Engenharia, PORTUGAL

## Abstract

The purpose of this study is to present the numerical investigations of an infection-based fractional-order nonlinear prey-predator system (FONPPS) using the stochastic procedures of the scaled conjugate gradient (SCG) along with the artificial neuron networks (ANNs), i.e., SCGNNs. The infection FONPPS is classified into three dynamics, susceptible density, infected prey, and predator population density. Three cases based on the fractional-order derivative have been numerically tested to solve the nonlinear infection-based disease. The data proportions are applied 75%, 10%, and 15% for training, validation, and testing to solve the infection FONPPS. The numerical representations are obtained through the stochastic SCGNNs to solve the infection FONPPS, and the Adams-Bashforth-Moulton scheme is implemented to compare the results. The infection FONPPS is numerically treated using the stochastic SCGNNs procedures to reduce the mean square error (MSE). To check the validity, consistency, exactness, competence, and capability of the proposed stochastic SCGNNs, the numerical performances using the error histograms (EHs), correlation, MSE, regression, and state transitions (STs) are also performed.

## 1. Introduction

Infectious diseases occur mainly when polluted foreign bodies like parasites, fungi, viruses, and germs enter the human body. The infection is transmitted into humans, animals, contaminated food, or contact with any ecological influences polluted with any individuals. There are many symptoms, severity and types of infectious viruses. The most common indications of these diseases in the body’s structure are pain, cough, fever, and flu [1,2]. Few diseases have minor symptoms that do not require any treatment or medication. Otherwise, numerous serious deathly diseases can disturb the population symmetry of numerous categories in the troposphere. The mathematical models are applied to forecast the species progression from the last few decades. The Lotka-Volterra systems [3,4] are used to avoid the numerous worst circumstances for numerous types as death was demonstrated. The Lotka-Volterra systems is used to avoid the numerous worst circumstances for numerous types as death was demonstrated. In recent years, researchers used this tool to expose the significance of a convinced strategy, which is applied by the administrations to switch few classes to measure as a substantial expedient [3,4].

The ecological-based models are considered more complicated for any form of infection that can distress the evolution of a few groups. In this work, the collaboration based on the predator-prey is observed. This contagion can distress the strength of the predator, and the hunting capability takes the predators in the situation of death. Recently, several applications have scrutinized the predator-prey interactions based on the incidence of communicable diseases [5–9]. Alternatively, some schemes reproduce the predators to achieve an operative hunt. The hunting relationship is known as one of the effective strategies of the predator, where many predators use it for hunting some target. This approach is implemented to reduce the hunting rate, and a few hunters perform in this procedure. The high competence rate of hunting is wild, lions, hyenas, and dogs. The first mathematical formulation is accessible based on the accurate predator performance, where a simple system was pragmatic to designate a collaboration [10]. To date, few investigations are based on the behavior of predator-prey interaction accessible in the references [11–14]. Consequently, the possessions of a communicable virus have been examined in the predator-prey statement along with the incidence of the association of the predator hunting.

A system of three species based on the prey population infection is measured, categorized into two modules, the susceptible prey and the infected. It is observed that time-fractional derivative has a variety of applications in order to designate the several forms of conditions, which is recognized by the remembrance of a dynamical system. The remembrance rate is recognized as a derivative order, and the memory function is known as the factional form of derivative. The derivative based on the time-fractional is executed to form the phenomena of numerous real-world systems [15,16]. The factional form of derivative based on the prey-predator systems has three compartments, which are mathematically presented along with the initial conditions (ICs) as [17]:

$$\left\{ \begin{array}{ll} D^{\nu }S(T)=-\delta I(T)S(T)+r\left(I(T)+S(T)\right)-\mu S(T)-\left(aP(T)+\lambda \right)S(T)P(T), & S(0)=i_{1},\\ D^{\nu }I(T)=-\left(\lambda +aP(T)\right)I(T)P(T)+\delta S(T)I(T)-\mu I(T), & I(0)=i_{2},\\ D^{\nu }P(T)=\left(I(T)+S(T)\right)e\left(aP(T)+\lambda \right)P(T)-mP(T), & P(0)=i_{3}, \end{array}\right.$$ (1)

Where the infected prey and susceptible are *I*(*T*), and *S*(*T*), whereas *P*(*T*) represents the predator population dynamics. The parameter *e* indicates the conversion rate of prey into predator biomass (infected or susceptible). The term *r* is the reproduction number of prey populations, and this infection does not directly transport vertically. It is like the mother-child sense, where the virus does not attack directly to the predator when prey is diseased. The term δ is the infection rate that shows the transmission rate in the population of the prey. The parameters (*aP*(*T*) + *λ*)*P*(*T*)*I*(*T*) and (*aP*(*T*) + *λ*)*P*(*T*)*S*(*T*) represent the functional forms based on the hunting collaboration [18]. *μ* represents the prey population death rate based on the natural mortality of the population of the predators. The ICs are *i*_1_, *i*_2_ and *i*_3_, respectively. The reputed analytical, numerical and power series solution are reported that can deal with the solution of system (1) using deterministic methodologies [19–32].

The novelty of this work is to present the numerical investigations of an infection-based fractional- order nonlinear prey-predator system (FONPPS) using the stochastic procedures of the scaled conjugate gradient (SCG) along with the artificial neuron networks (ANNs), i.e., SCGNNs. The stochastic SCGNNs procedures have never been applied to solve the biological-based model based on the infection FONPPS. Three different variations based on the sample information, authentication, testing, and training have been applied. The data proportions are applied 75%, 10, and 15% for training, validation, and testing to solve these infection FONPPS. The numerical performances are presented through the stochastic SCGNNs to solve the infection FONPPS, and the comparative investigations have been performed using the reference solutions based on the Adams-Bashforth-Moulton method. The stochastic computing techniques can solve and perform numerical results based on nonlinear and complex problems [33–36]. These investigations based on integer order inspired the authors to solve the equation’s fractional-order nonlinear mathematical system. Hence the authors selected a well-known application of the biological system, i.e., infection FONPPS, a fractional-order differential system of nonlinear equations, and numerical soundings of this complex system have been presented using the SCGNNs. Some novel features of the present study are discussed as:

- The numerical investigations based on the fractional order nonlinear mathematical system of equations are addressed using the stochastic procedures.
- A novel design of the artificial neural network along with the scaled conjugate gradient is applied first time to solve the biological system based on the infection FONPPS.
- The numerical investigations of the three different cases based on the fractional order are presented for solving the infection FONPPS.
- The comparison of the results has been presented using the Adams-Bashforth-Moulton method.
- The small absolute error (AE) authenticates the performances of the proposed stochastic SCGNNs scheme for solving the infection FONPPS.
- A good performance of the regression, mean square error (MSE), correlation, state transitions (STs) and error histograms (EHs) validate the consistency and reliability of the proposed SCGNNs scheme for solving the infection FONPPS.

The remaining parts of the paper are provided as follows: The SCGNNs methodology design is presented in Section 2. Section 3 describes the numerical solutions of the infection FONPPS using the SCGNNs scheme. The final remarks are stated in Sect 4.

## 2. Methodology: SCGNNs

**Fig 1:**
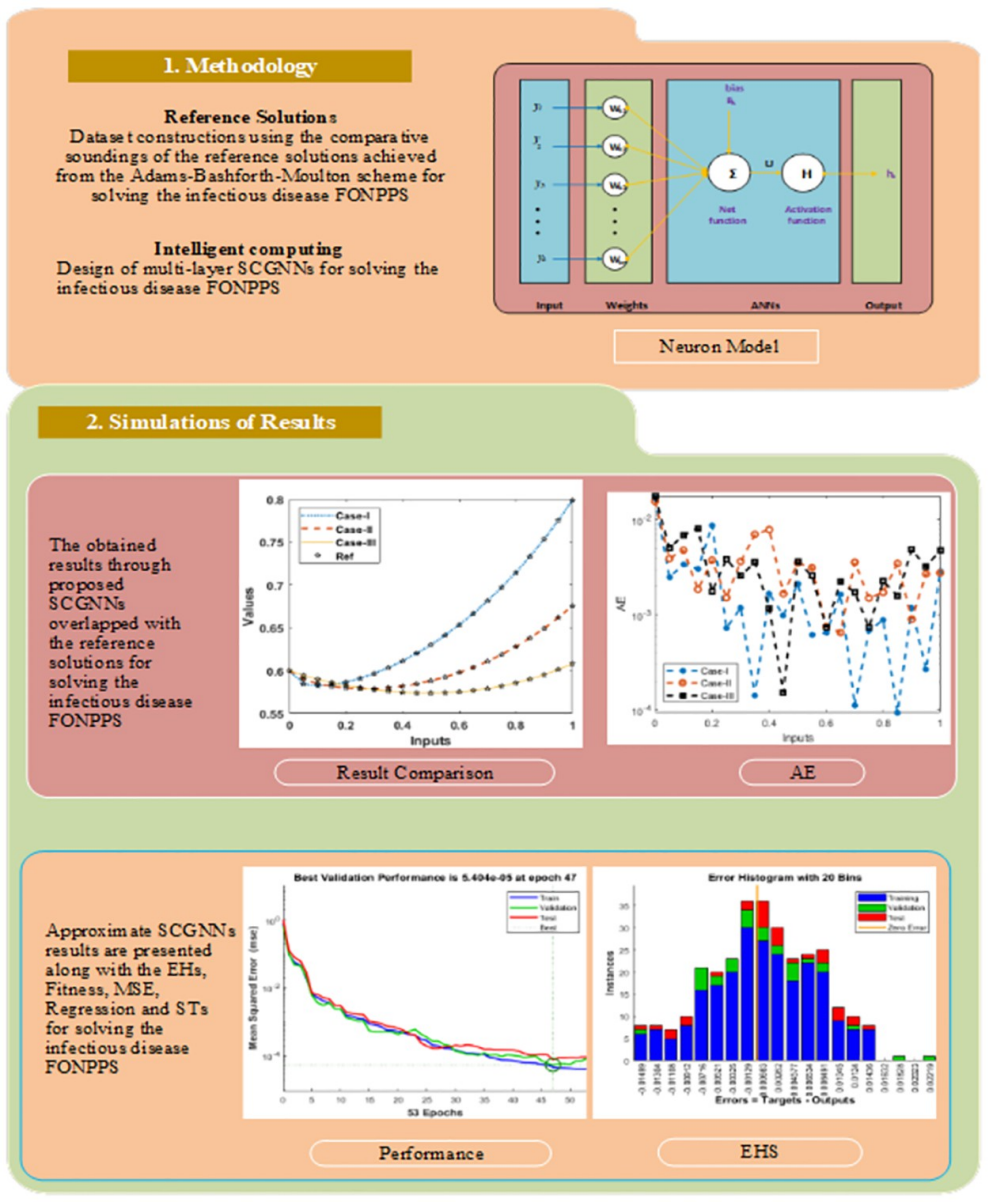
Workflow diagram of the proposed SCGNNs for solving the infection FONPPS.

**Fig 2:**
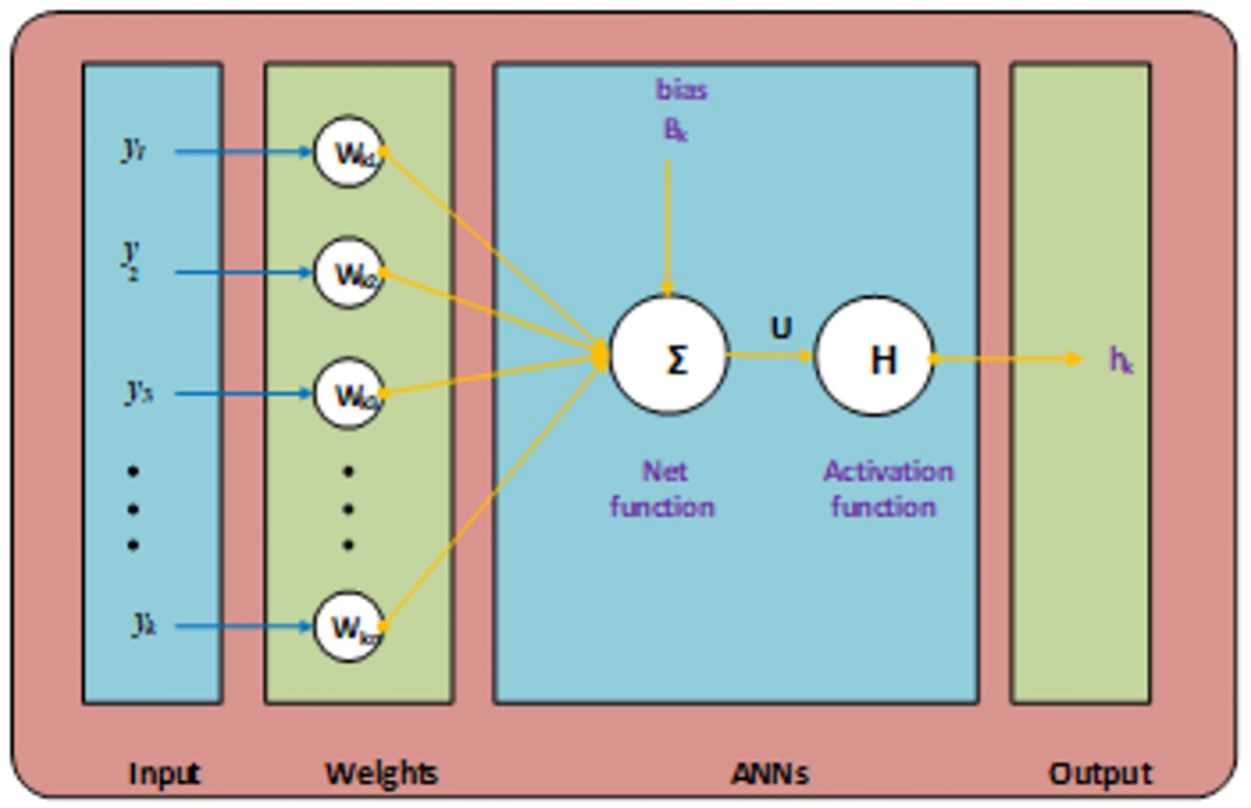
Proposed structure for a single neuron.

The current section shows the proposed SCGNNs methodology is presented for solving the infectious disease FONPPS. The designed scheme SCGNNs is classified in two phases. The essential trials of the computational stochastic SCGNNs are given in the first phase, while the execution procedure of the stochastic computing approach is provided to solve the infectious disease SCGNNs. An appropriate optimization process based SCGNNs is provided in Fig 1 along with the multi-layer routines, although to present a single neuron, the proposed scheme is plotted in Fig 2. The computing-based stochastic measures are implemented using the ‘nftool’ command, a build-in procedure in MATLAB. The data proportions are applied 75%, 10, and 15% for training, validation, and testing to solve the infection FONPPS.

## 3. Numerical performances of the infection FONPPS

**Fig 3:**
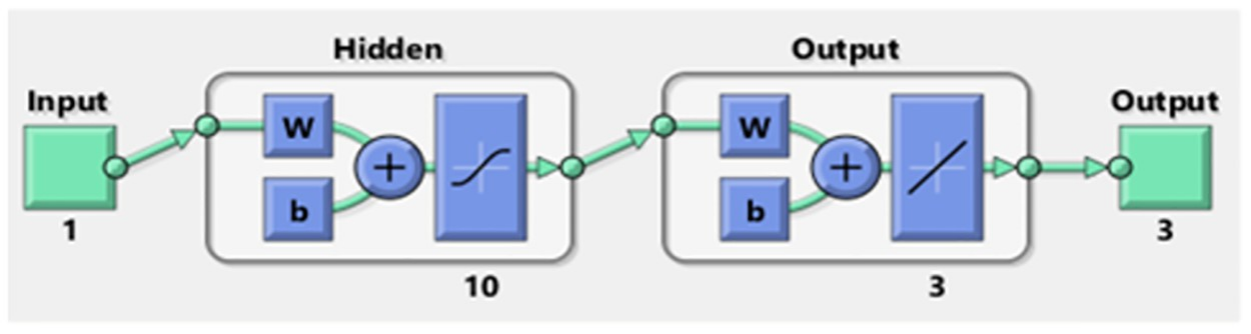
Proposed SCGNNs for solving the infection FONPPS.

In this section, the numerical procedures of the infection FONPPS are presented by using the proposed SCGNNs. The literature values to solve the infection FONPPS are *λ* = 0.5, *r* = 1.5, *m* = 0.5, *a* = 0.5, *μ* = 0.5, *δ* = 0.5, *e* = 0.5, *i*_1_ = 0.2, *i*_2_ = 0.7, and *i*_3_ = 0.6. Three cases of the infection FONPPS based on the fractional-order, i.e., *ν* = 0.5, 0.7, and 0.9 have been provided. The complete results have been accomplished for each type of infection FONPPS lie around [0, 1] with the smallest step size, i.e., 0.01. The neurons have been taken as ten throughout this study, and the data proportions are applied 75%, 10, and 15% for training, validation, and testing to solve these cases of the infection FONPPS. The obtained numerical performances using the 10 number of neurons based on the SCGNNs for solving the infection FONPPS are illustrated in Fig 3.

**Fig 4:**
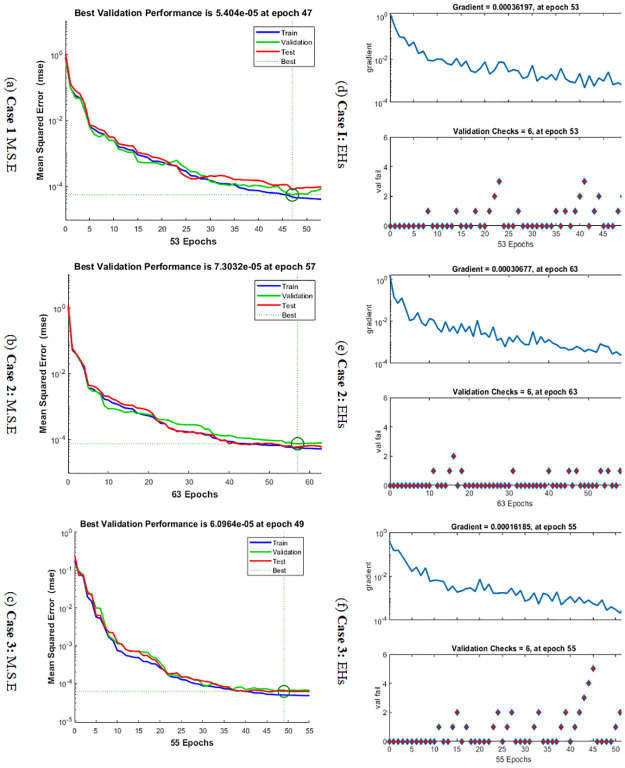
Performances of MSE (a-c) and STs values (d-f) for solving the infection FONPPS. (a) **Case 1** M.S.E (b) **Case 2**: M.S.E (c) **Case 3**: M.S.E (d) **Case I**: EHs (e) **Case 2**: EHs (f) **Case 3**: EHs.

**Fig 5:**
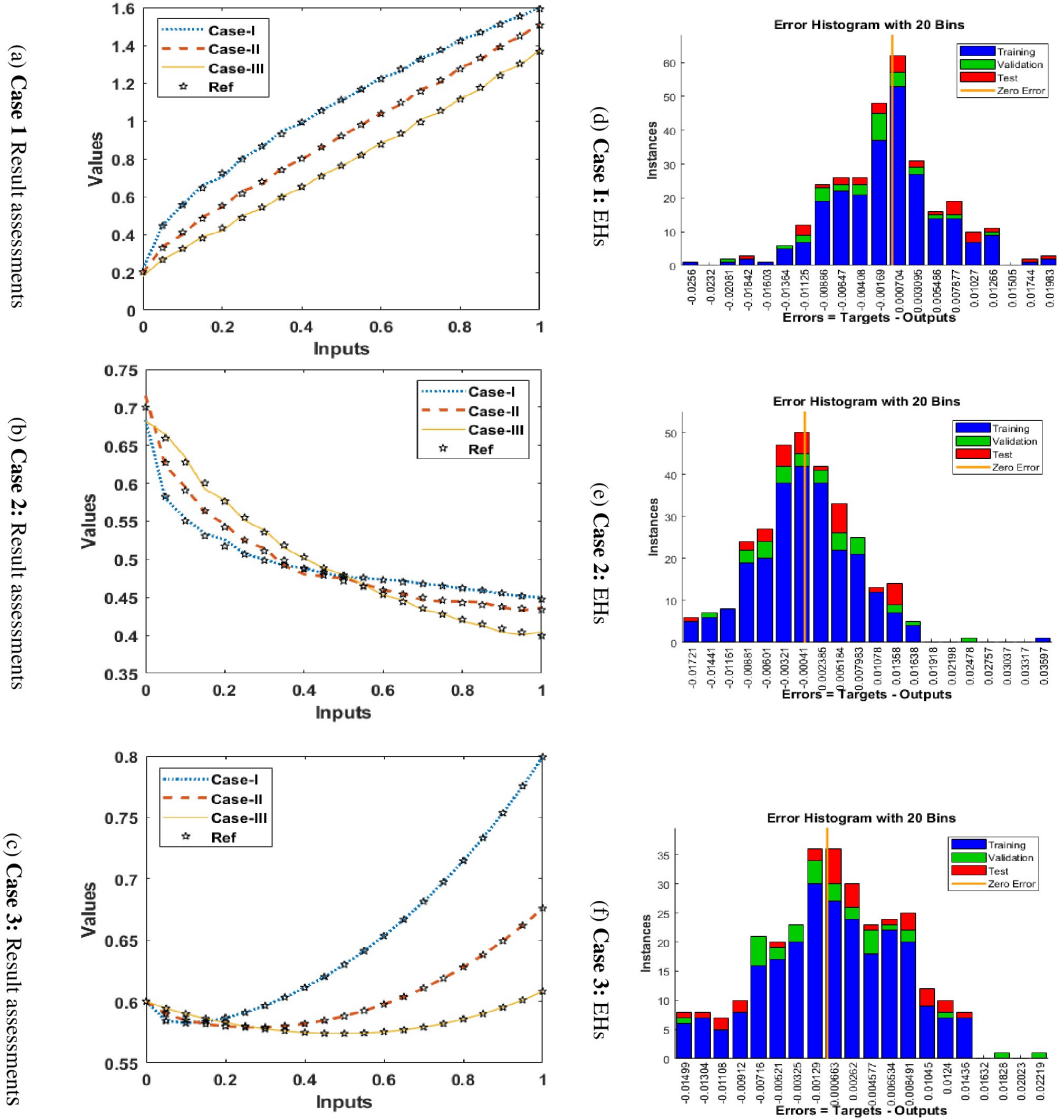
Results valuations (a-c) and EHs values (d-f) for solving the infection FONPPS. (a) **Case 1** Result assessments (b) **Case 2**: Result assessments (c) **Case 3**: Result assessments (d) **Case I**: EHs (e) **Case 2**: EHs (f) **Case 3**: EHs.

**Fig 6:**
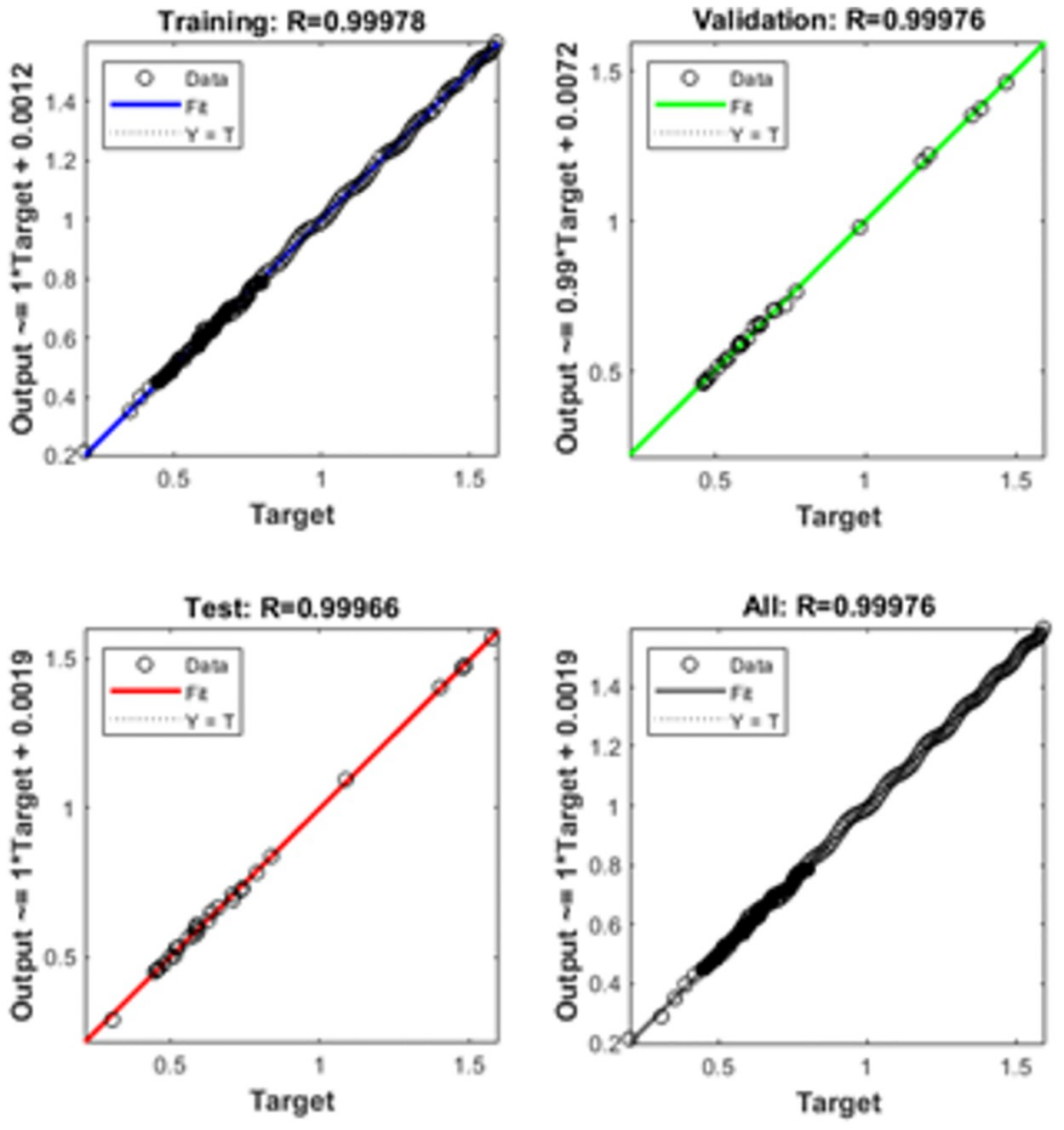
Regression actions for solving the infection FONPPS of Case 1.

**Fig 7:**
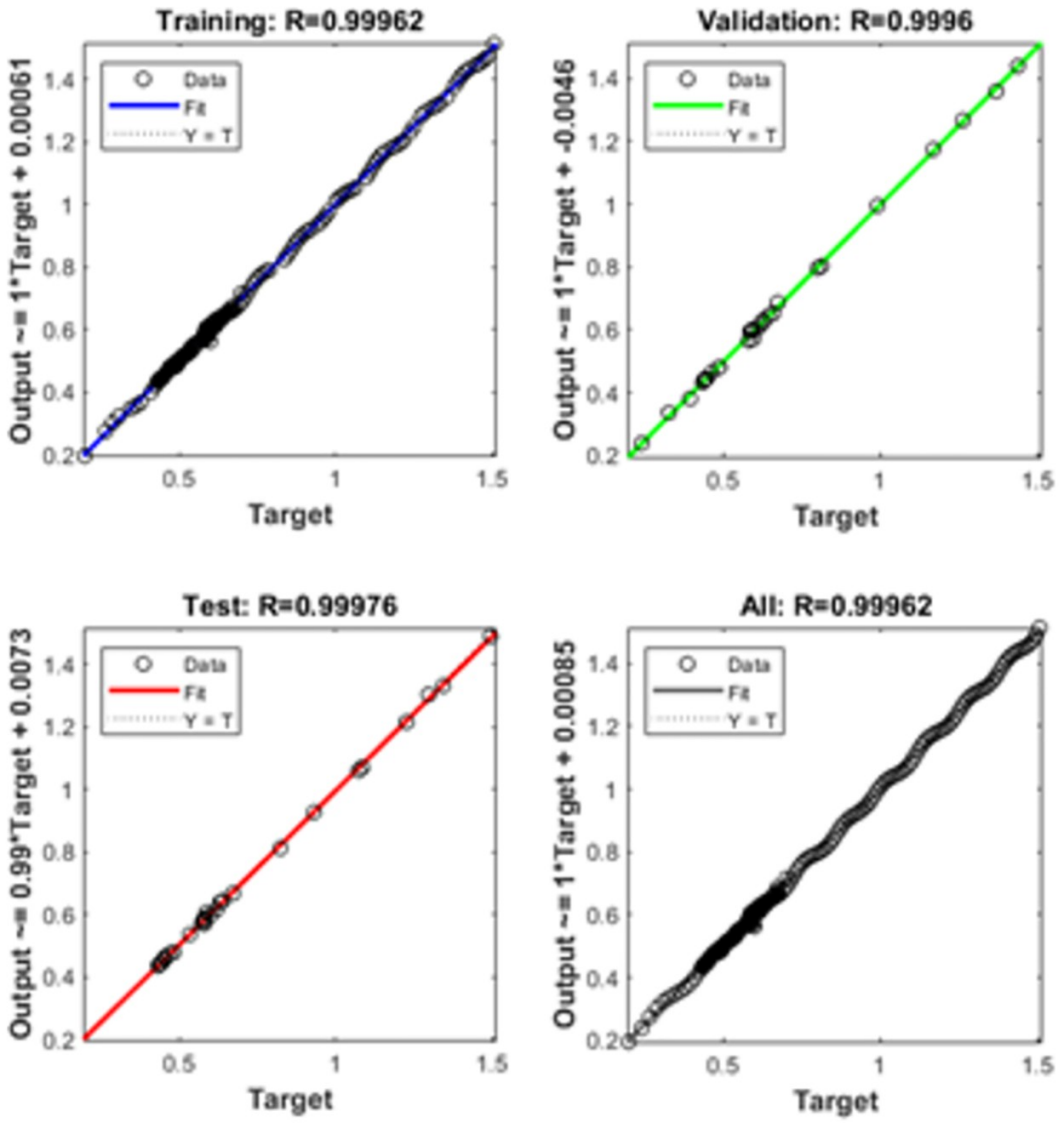
Regression actions for solving the infection FONPPS of Case 2.

**Fig 8:**
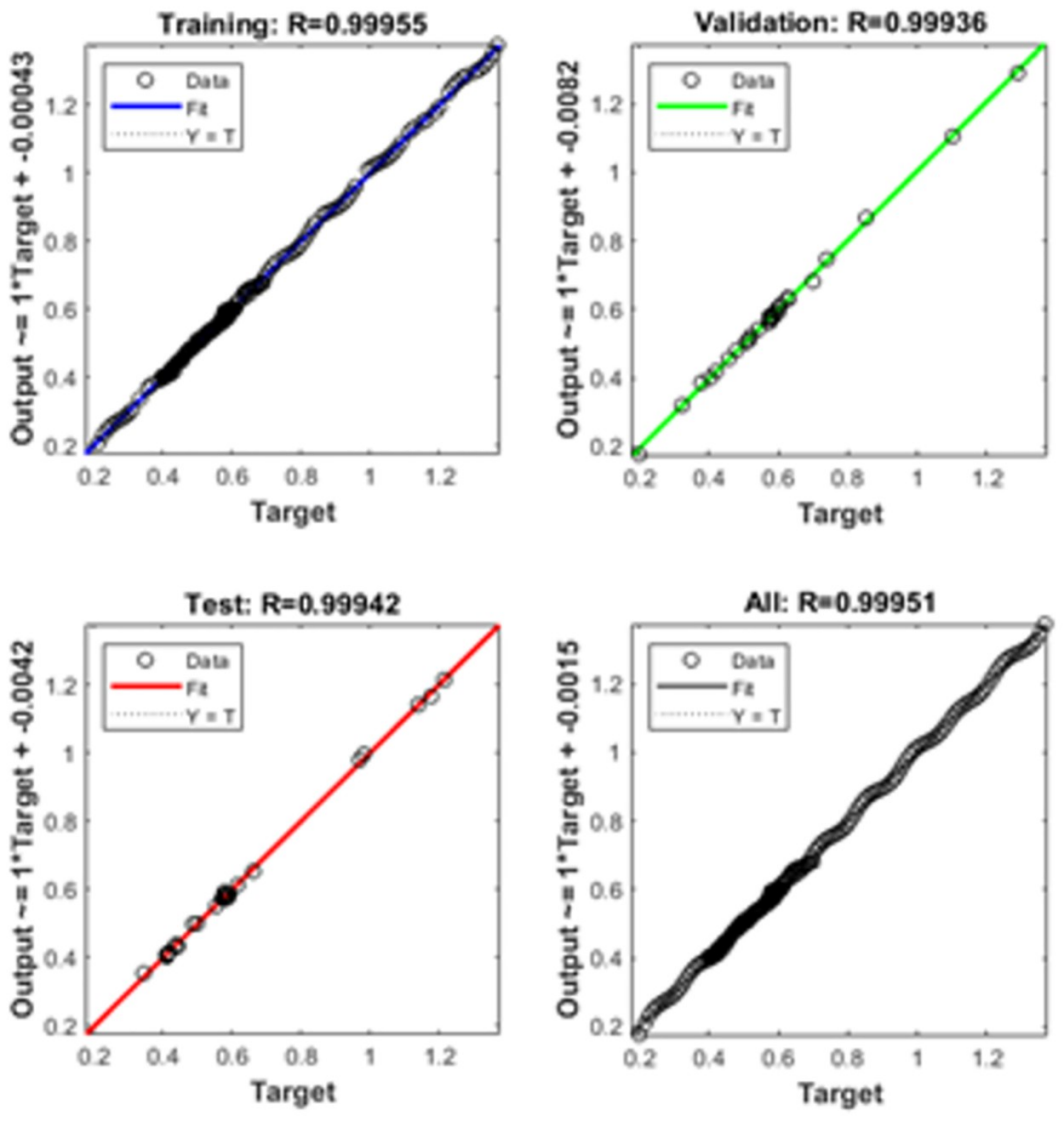
Regression actions for solving the infection FONPPS of Case 3.

The graphical representations using the proposed SCGNNs scheme for solving the infection FONPPS are presented in Figs 4–8. The performances based on the MSE and STs for solving the infection FONPPS are illustrated in Fig 4. The MSE values for the training, best curves states, authentication as well as testing are illustrated in (Fig 4a–4c), while the best values of the STs values for solving the infection FONPPS are illustrated in (Fig 4d–4f) at epochs 47, 57 and 49, respectively. The attained performances occur at 5.404× 10^-05^, 7.3032 × 10^-05^, and 6.0964 × 10^-05^, respectively. The values through the gradient based on the proposed SCGNNs for solving the infection FONPPS are calculated 3.6197× 10^−04^, 3.0677 × 10^−04^ and 1.6185 × 10^−04^, respectively. The values drawn in these figures represent the accuracy, precision, and convergence of the proposed SCGNNs for solving the infection FONPPS. The fitting curve plots have been illustrated in (Fig 5a–5c) for solving the infection FONPPS using the proposed SCGNNs. These illustrations have been drawn based on the comparison performances of the obtained outcomes through the proposed SCGNNs. (Fig 5d–5f) are drawn based on the EHs values, which lie in the 7.04 × 10^-04^, 4.1 × 10^-04^, and 6.63 × 10^-04^ for cases 1, 2, and 3, respectively. The regression performances are illustrated in Figs 6–8 t for solving the infection FONPPS. These correlation values prove the regression performances around one that indicates the perfect system. The plots using the performances of testing, training and authentication perform the accuracy of the proposed SCGNNs for solving the infection FONPPS. Moreover, the convergence using the MSE measure with the calculations of the epochs, complexity, training, substantiation, testing, and backpropagation presentations is provided in Table 1 for solving the infection FONPPS using the proposed SCGNNs.

**Table 1:** Statistical procedures for solving the infection FONPPS using the proposed SCGNNs.

Case	M.S.E			Performance	Gradient	Epoch	Time
	[Training]	[Validation]	[Testing]				
1	4.67 × 10^−05^	5.40 ×10^−05^	7.75 × 10^−05^	3.97×10^−05^	3.62 × 10^−04^	53	5
2	5.46 × 10^−05^	7.30 ×10^−05^	5.87 × 10^−05^	5.13×10^−05^	3.05 × 10^−04^	63	5
3	4.19× 10^−05^	6.09 × 10^−05^	6.48 × 10^−05^	4.74×10^−05^	1.62 × 10^−04^	55	2

**Fig 9:**
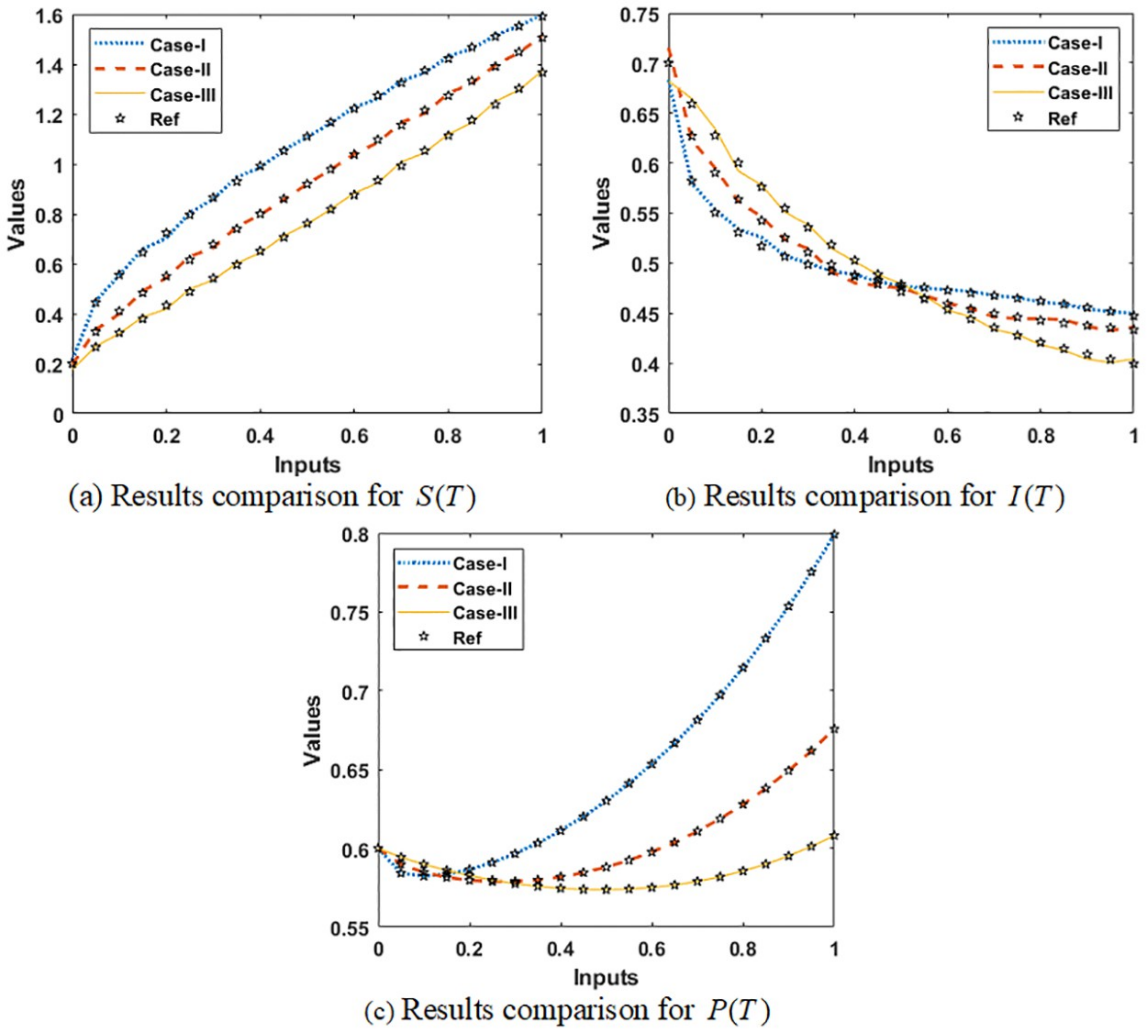
Result comparison illustrations for solving the infection FONPPS. (a) Results comparison for *S*(*T*) (b) Results comparison for *I*(*T*) (c) Results comparison for *P*(*T*).

**Fig 10:**
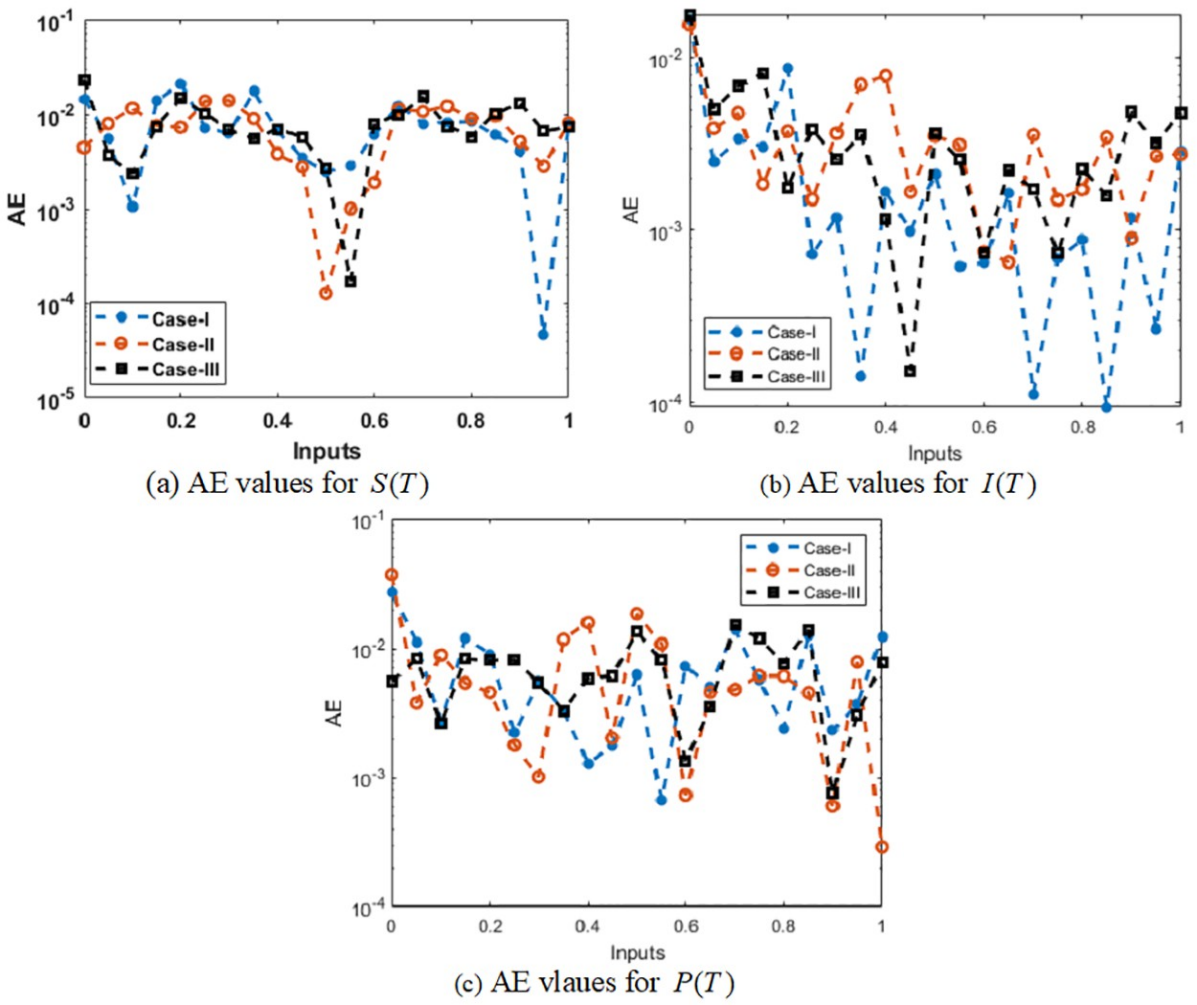
Values of the AE for solving the infection FONPPS. (a) AE values for *S*(*T*) (b) AE values for *I*(*T*) (c) AE vlaues for *P*(*T*).

The AE values using the comparative measures are demonstrated in Figs 9 and 10 for solving the infection FONPPS. The numerical measures for each class of the infection FONPPS using the proposed SCGNNs. The results for each the infection FONPPS are accessible using the proposed SCGNNs are specified in Fig 9(a)–9(c). One can find that the proposed and reference solutions overlapped for each class of the infection FONPPS. These matching of the results signify the correctness and precision of the proposed SCGNNs for each class of the infection FONPPS. The AE procedures for each class of the infection FONPPS are illustrated in Fig 10(a)–10(c). The values of the AE for *S*(*T*) based on the infection FONPPS are considered around 10^−02^ to 10^−05^ for case 1, while the AE values for cases 2 and 3 are calculated 10^−02^ to 10^−04^. The AE performances for *I*(*T*) based on the infection FONPPS lie around 10^−03^ to 10^−04^ for case 1, while the AE values for cases 2 and 3 are calculated 10^−02^ to 10^−04^. The values of the AE for *P*(*T*) based on the infection FONPPS lie around 10^−02^ to 10^−04^ for cases 1, 2 and 3.

## 5. Conclusions

The current investigations present the numerical investigations of an infection-based fractional order nonlinear prey-predator system using the stochastic procedures of the scaled conjugate gradient and the artificial neuron networks. The stochastic computational procedure SCGNNs is applied to solve three different cases using different fractional-order values. The data proportions applied 75%, 10, and 15% for training, validation, and testing to solve the infection FONPPS. Ten numbers of neurons have been used to solve the nonlinear biological-based differential model. The numerical simulations of the infectious disease FONPPS are accomplished using the SCGNNs, while the competitive performances have been presented using the Adams-Bashforth-Moulton approach. The numerical results of the nonlinear fractional-order biological system are calculated using the computational SCGNNs to reduce the mean square error. To ratify the exactness, reliability, capability, and aptitude of the proposed SCGNNs, the numerical measures are plotted using the regression, MSE, STs, correlation, and Ehs. The identical performances designate the precision and accuracy of the proposed stochastic scheme and the AE values found in suitable ranges based on the nonlinear fractional-order biological system. The AE values, and the plots of other performances represent the dependability and consistency of the proposed approach. In future studies, the stochastic SCGNNs are pragmatic to achieve the results of the lonngren-wave systems and fractional order nonlinear systems [37–45].
